# Bulked segregant transcriptome analysis in pea identifies key expression markers for resistance to *Peyronellaea pinodes*

**DOI:** 10.1038/s41598-022-22621-2

**Published:** 2022-10-28

**Authors:** Sara Fondevilla, Nicolas Krezdorn, Diego Rubiales, Björn Rotter, Peter Winter

**Affiliations:** 1 Institute for Sustainable Agriculture, CSIC, 14004 Córdoba, Spain; 2 GenXPro GmbH, 60438 Frankfurt Am Main, Germany

**Keywords:** Genetics, Plant sciences

## Abstract

*Peyronellaea pinodes* is a devastating pathogen of pea crop. Quantitative trait loci (QTL) associated with resistance have been identified, as well as genes differentially expressed between resistant and susceptible pea lines. The key question is which of these many genes located into these QTLs, or differentially expressed, are the key genes that distinguish resistant from susceptible plants and could be used as markers. To identify these key genes, in the present study we applied MACE (Massive Analysis of cDNA Ends) -Seq to a whole Recombinant Inbred Line population segregating for resistance to this disease and their parental lines and identified those genes which expression was more correlated with the level of resistance. We also compared gene expression profiles between the most resistant and the most susceptible families of the RIL population. A total of 6780 transcripts were differentially expressed between the parental lines after inoculation. Of them, 803 showed the same expression pattern in the bulks formed by the most resistant and most susceptible RIL families. These genes, showing a consistent expression pattern, could be used as expression markers to distinguish resistant from susceptible plants. The analysis of these genes also discovered the crucial mechanisms acting against *P. pinodes*.

## Introduction

Ascochyta blight is the most relevant foliar pea disease. It is incited by a complex of fungi, being *Peyronellaea pinodes* the most common and harmful pathogen^1^.

*P. pinodes* is a hemibiotrophic fungus. When conditions are suitable and the host is susceptible, *P. pinodes* conidial infection starts with the production of a germ tube. *P. pinodes* penetrates directly through the cuticle to epidermal cells. Once penetrated, hyphae grow along the extracellular space. During this first phases of the infection, hyphae grow inside the host cell walls by moving the periplasmatic cell content without protoplast collapse or killing of epidermal cells. This biotrophic phase is followed by a necrotrophic phase in which the fungus colonizes the mesophyll producing necrotic lesions^2^.

Resistance to *P. pinodes* in pea is a polygenic character and several efforts to map the resistance^3–9^ identified genomic regions associated with resistance, but failed to reveal the genes underlying the observed QTL. Therefore, as an alternative to genetic mapping, we applied comparative gene expression analyses between inoculated and non-inoculated susceptible and partial resistant plants to gain an understanding of physiological mechanisms underlying resistance, with the aim to directly identify the genes involved. On this way, in a previous study, we performed transcriptome profiling of the resistant pea accession P665 and the susceptible cultivar Messire, inoculated with *P. pinodes*, using a heterologous microarray carrying 70 mer oligonucleotides representing *M. truncatula* genes. In this study 346 genes differentially regulated between P665 and Messire were identified^10^. Later then, using the SuperSAGE technique, we identified 509 pea genes which expression was induced or repressed in accession P665 when infected by *P. pinodes*^11^.

Though these early studies gave useful insights into fundamental responses of the plant upon infection with *P. pinoides*, they failed to reveal putative marker genes useful for breeding. To solve this problem, in the present study we chose an experimental set-up, that differs from these efforts in several aspects. First, we not only profiled the transcriptome of a resistant and susceptible accession, as before, but also that of an inoculated Recombinant Inbred Line (RIL) population segregating for resistance. Moreover, while in the microarray experiment^10^ only a resistant and a susceptible line infected with *P. pinodes* were analysed, and in the SuperSAGE experiment only the inoculated and non-inoculated resistant accession was studied, in the present study we included all treatments (inoculated and non-inoculated plants from the resistant and the susceptible accessions) in the analysis. Secondly, we exploited MACE (Massive Analysis of cDNA Ends -Seq^12^), technique enabling to produce more comprehensive, genome-wide transcription profiles at reasonable costs than the methods used before. In pea, MACE-Seq has been successfully applied for evolutionary studies^13^, identification of SNP-based genic markers^14^ and mutant symbiotic alleles^15^ as well as for gene expression profiling analyses for pant specific traits^16^.

Here we apply MACE-Seq for transcriptome profiling of the parental lines and a whole RIL population segregating for resistance. This allowed us to correlate the expression of each of more than 13,700 expressed transcripts with the level of resistance. Furthermore, with the analysis of the most contrasting bulks of the RIL population we performed what we call “bulk segregant transcriptome analysis” (BSTA) to compare gene expression profiles between the most resistant and the most susceptible families of the RIL population. This permitted us to unravel putative key processes and their underlying genes that distinguish resistant from susceptible plants. qRT-PCR markers derived from these genes may be used as innovative expression markers in Marker-assisted Selection (MAS).

## Materials and methods

All experiments were performed with accordance to relevant regulations and guidelines.

### Plant material

Two genotypes, P665 and Messire, as well as a F_2:6_ RIL population obtained from the cross between these accessions were used in the experiments. Cultivar Messire (*Pisum sativum* ssp. *sativum*) is highly susceptible to *P. pinodes*. P665 is a *P*. *sativum* ssp. *syriacum* (a wild relative of pea) accession that shows incomplete resistance to this pathogen^17^.

### Inoculation and sampling

To discern which could be the most suitable time points for sampling of material for transcriptomic analysis, we first carried out a histological study in the same conditions as those to be used in the transcriptomic study and observed the time course of the infection. Plants of P665 and Messire were inoculated with *P*. *pinodes* as described below and leave samples were observed at 12, 24, 36 y 48 hai (hours after inoculation). At these time points, the percentage of germinated spores, the penetration into the epidermis and the formation of necrotic lesions in the mesophyll were quantified in four leaflets per genotype and time point as described in^18^. Previous histological studies^19^, indicated that resistance of P665 is due to the presence of a mechanisms stopping *P. pinodes* at the epidermis and a further mechanisms limiting its growth in the mesophyll. Consequently, the times points that, according to the results obtained in the present study, corresponded to penetration of pea epidermis by *P*. *pinodes* (24 h) and appearance of necrotic lesions in the mesophyll caused by *P. pinodes* (48 h), were selected as the most suitable times to harvest the samples.

The experiments were carried out in three independent replicates. A diagram of the sampling design followed in each of the three replicates is shown in Supplementary Fig. S1. In the experiment involving P665 and Messire, nine plants of Messire and 27 plants of accession P665 per replicate were inoculated. More plants were included from P665 than from Messire because the leaves of P665 are small, and so, more plants were needed to obtain enough plant material for RNA extraction. Three plants from Messire and 12 plants from P665 were used for leaves sampling at each time point while three of each genotype were used for disease symptoms scoring 7 hai, using the scale described by Roger and Tivoli^20^. For inoculation, Co-99, a monoconidial isolate derived from an isolate obtained from ascochyta blight infected pea material collected in pea fields at Córdoba (Spain) during 1999 was used. The isolate was grown in Petri dishes containing PDA medium at 23 °C, exposed to a 16 h photoperiod. A spore suspension of 5 × 10^5^ spores per milliliter was obtained as described in^11^ and applied to the third and fourth leaf of plants at 5–6 leaf stage using a paintbrush. Then, plants were maintained at high humidity and darkness during 24 h by using ultrasonic humidifiers. In addition, per replicate and time point, three Messire and 12 P665 control, non-inoculated plants, were inoculated with sterile water and maintained in the same environmental conditions as inoculated plants. Afterward, plants were transferred to a growth chamber (12 h light/12 h darkness photoperiod, at 250 μmolm^-2^ s^-1^).

In the experiment involving P665 and Messire, leaves (3rd and 4th) from inoculated and control plants were cut 24 and 48 h after inoculation (hai), frozen in liquid nitrogen and stored at − 80 °C until RNA extraction. In the case of the experiment using the RIL population P665 × Messire, 96 families from this population were inoculated as reported above, excepting that in this case also the fifth leaf was inoculated. Messire and P665 plants were also included to check that the inoculation and disease development progressed well. Samples were taken from the third and fourth leaves 24 hai, while the evaluation of the disease was evaluated in the fifth leaf 7 dai. Two plants represented each family in each replicate.

### RNA extraction, preparation and sequencing of MACE libraries

In the case of the experiment including P665 and Messire, RNA from each replicate of the different treatments was extracted separately. In the case of the experiment including the RIL population, the replicates were pooled. RNA was extracted using TRISure (Bioline, London, UK) according to the manufacturer’s protocol and treated with RQ1 RNase-Free Dnase (Promega, Madison, USA) to eliminate any potential residual DNA. Quantity and purity of total RNA was analysed using a NanoDropND1000 (NanoDropTechnologies, Inc.,Wilmington, USA) and its integrity was examined on agarose gels.

Around 5 μg of total RNA was used to construct MACE libraries using the “MACE-Kit” (GenXPro GmbH, Frankfurt am Main, Germany) following the supplier’s manual. MACE technique analyses 3′- cDNA ends of 300–500 bp long, fragmented cDNA. The fragments generated are sequenced from their 5′-ends. In that way only one sequence from each transcript is produced. The potential bias produced during the PCR amplifications included in the preparation of the libraries was avoided using GenXPro’s PCR-bias-proof technology “TrueQuant”, included in the MACE kit, that allows distinguishing PCR copies from original transcripts. Sequencing (94-bp tags from each library) was done on an Illumina HiSeq2500 machine (Illumina, Inc., San Diego, CA, USA).

### Reads preprocessing and mapping

Sequence reads obtained were further analysed using GenXPro’s MACE analysis pipeline. In summary, libraries were sorted according to their respective index. Later on, duplicates obtained during PCR amplifications were identified and eliminated by TrueQuant technology. In addition, reads were filtered for quality and homopolymer. In addition, sequencing adapter primers and cDNA synthesis primers were eliminated.

To distinguish *Pisum sativum* sequences from those coming from *P. pinodes*, reads were mapped against the *Pisum sativum* transcriptome reported by Alves-Carvalho et al.^21^. However, when we mapped our reads to this transcriptome, we noticed that a significant part of the reads, including those coming from control samples, could not be assigned to this reference. As the transcriptome used as reference was from *P. sativum* ssp. *sativum* and did not include any pathogen infection treatment, these unmapped reads could correspond to reads specific for *P. sativum* ssp. *syriacum* or specific for plant-pathogen interactions. Consequently, in order to enrich our reference, we assembled these reads that did not match this transcriptome (*de-novo* assembly), using trinityrnaseq-2.2.0. Contigs coming from control plants were considered to belong to the plant. In addition, to select plant contigs from the inoculated samples that may be only expressed during infection, we performed a blastn homology search in the whole nucleotide collection (NT) from the NCBI data base (https://www.ncbi.nlm.nih.gov/home/download/). Contigs that matched plants were extracted and added to our reference. After that, we combined the sequences from the *P. sativum* transcriptome^21^ with our contigs assigned to the plant and used CAP3 for reducing very similar transcripts. To further reduce redundancies, we used cd-hit software^22^ considering 90% identity. To annotate the contigs, those contigs not matching the *P. sativum* transcriptome^21^ were annotated to the UniProtKB/Swiss-Prot, UniProtKB Fabaceae proteins databases by the BlastX tool (http://www.ncbi.nlm.nih.gov).

### Identification of transcripts differentially expressed between treatments

For the identification of transcripts differentially expressed between treatments we used the custom plant reference described above. First, all transcripts having less than 10 reads were eliminated. Then a custom workflow was used to calculate frequencies. Briefly, MACE reads were mapped to our plant reference using the SOAP2.2 software^23^ allowing 5 base mismatches. DEseq2 package^24^ was used for normalization and differential expression analysis. To identify transcripts over-expressed during treatment “A”, compared to treatment “B”, normalized expression values were compared between “A” libraries and “B” libraries. We considered a transcript to be up-regulated when the normalized expression value of the transcript was statistically significantly different (p_adjusted_ ≤ 0.05), and at least 2 times higher, in treatment “A” compared to treatment “B” for at least one time point after inoculation, or when the transcript was only present in treatment “A” libraries. Similarly, a gene was considered to be down-regulated in treatment “A” compared to treatment “B” when the normalized expression value of the transcript was statistically significantly different (p_adjusted_ ≤ 0.05), and at least 2 times lower in treatment “A” compared to treatment “B” for at least one time point after inoculation, or when the transcript was only present in treatment “B” libraries. Several comparisons were made. Thus, we compared transcript frequencies between P665 and Messire inoculated plants, at 24 or 48 hai, and between P665 and Messire control non-inoculated plants. In addition, we also identified the genes differentially expressed between the eight more resistant families (called resistant bulk in this article) and the eight more susceptible families (called susceptible bulk in this article) derived from the RIL population P665 × Messire. To select the most resistant and the most susceptible families we took into account the disease evaluation performed in this experiment as well as previous disease evaluations carried out in this population under control and field conditions^6^.

### Analysis of correlation between transcript expressions and the level of resistance

To analyse the level of correlation between the expression of each transcript and the level of resistance to *P. pinodes*, we calculated Pearson’s correlation coefficient between the normalized expression value of the transcripts in each RIL family and the disease rating obtained for these families in the evaluation carried out in the experiment described in this article. In order to obtain accurate conclusions, only genes for which expression was detected in at least 50 RIL families were included in the analysis.

### Go-enrichment test

The percentage of transcript belonging to each GO Slim category was compared between the different subsets of transcripts and all the transcripts included in our plant reference using χ^2^ statistics in order to identify the GO slim terms that were over- or under-represented in these subsets.

### Validation of expression profiles obtained by MACE by qRT-PCR

The expression trends of 10 genes with a putative relevant role in resistance to *P. pinodes*, that, in our MACE-Seq analysis, were significantly stronger expressed in P665 inoculated plants, compared to Messire inoculated plants, as well as in the bulk of resistant RIL families compared to the bulk of susceptible RIL families, were examined with two-step qRT-PCR. In the case of P665 and Messire, qRT-PCR were carried out for each of the three replicates, while in the case of the resistant/susceptible bulks equal amount of RNA from each family forming the bulk was pooled.

From each RNA sample, total RNA (5 μg) was reverse-transcribed with the SuperScript IV First-Strand Synthesis System (Invitrogen, Karlsruhe, Germany). SYBR Green was used to monitor dsDNA synthesis and polymerase chain reactions were carried out in a 48-well plate with a StepOne Real Time PCR System (Applied Biosystems, Foster City, CA, USA). Reaction mix (10 μl in total) included 5 μl Fast Start Universal SYBR Green Master (ROX), 1 μl cDNA, and 0.3 μM of each primer. The standard thermal profile followed was: polymerase activation (95 °C for 10 min), amplification and quantification (40 cycles; 95 °C for 15 s, 60 °C for 1 min) and dissociation curve generation (95 °C 15 s, 60 °C 1 min, 95 °C 30 s).

To design primers we used Probe Finder 2.53 (Universal Probe Library, Roche). The genes selected and the sequence of the primers designed to amplify them are included in Supplementary Table 1. For normalization, TUB, and GAPDH genes^25^ were used.

The PCR efficiency of each primer pair in each individual reaction was calculated using LingRegPCR 7.5 software and used to calculate an average efficiency (E) per primer pair. This average efficiency was used to calculate the expression in each reaction using the formula Expression = E^CT^. A normalization index was calculated for each plate as the geometric mean of the expression of the reference genes and a relative expression was calculated for each reaction as the ratio of the gene expression of the gene of interest in each reaction against the normalization index.

## Results

### Disease assessment

Confirming previous studies, accession P665 displayed incomplete resistance to *P. pinodes*, showing one week after inoculation an average disease rating of 2.9 in the 0–5 scale described by Roger and Tivoli^20^. Messire was highly susceptible showing an average disease rating of 4.8. The RIL (P665 × Messire) population showed a continuous distribution with disease rating from 2 to 5 (Supplementary Fig. S2).

### Identification of genes differentially expressed between P665 and Messire

After eliminating duplicates and low quality reads a total of 160,681,582 sequencing reads from Messsire and P665 were processed, with an average of 6,695,077 reads per library (Table 1).

**Table 1:** Summary of reads counts.

Accession	Treatment	Time point	Reads after filtering
Messire	Control	24 hai	16,858,847
Messire	Control	48 hai	19,313,611
Messire	Inoculated	24 hai	21,557,718
Messire	Inoculated	48hai	24,175,905
P665	Control	24 hai	17,725,253
P665	Control	48 hai	18,555,014
P665	Inoculated	24 hai	22,174,341
P665	Inoculated	48 hai	20,321,181
RIL population	Inoculated	24 hai	566,451,353

*hai* = hour after inoculation with *P. pinodes*.

**Figure 1:**
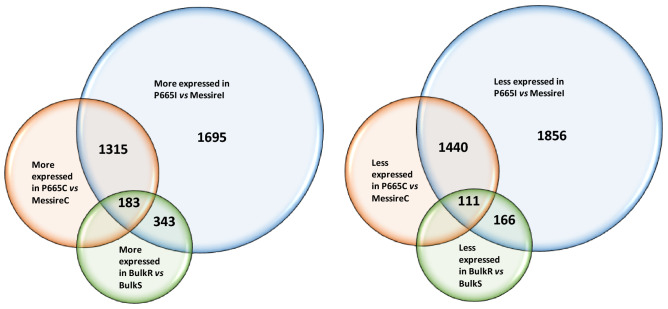
Venn diagram showing the number of genes in each expression pattern category. *P665I* = P665 inoculated plants; *MessireI* = Messire inoculated plants, *P665C* = P665 control plants, *MessireC* = Messire control plants, *BulkR* = bulk of the most resistant RIL families, *BulkS* = bulk of the most susceptible RIL families.

Reads were obtained for 27,805 transcripts of which 21,773 had at least 10 reads and were included in the subsequent analyses. In order to focus on genes that could have a more relevant role in the resistance response against *P. pinodes*, we first identified those genes that were differentially expressed between Messire and P665 inoculated plants. A total of 6780 transcripts were differentially expressed between P665 and Messire inoculated plants in at least onetime point after inoculation (Supplementary Data 1). Subsequently we classified these genes in four categories: (1) genes up-regulated in P665 vs. Messire inoculated plants and also between P665 and Messire control plants (called constitutively up-regulated in this article), (2) genes up-regulated in P665 vs. Messire inoculated plants but not between P665 and Messire control plants (called induced after infection in this article), (3) genes down-regulated in P665 vs. Messire inoculated plants and also between P665 and Messire control plants (called constitutively down-regulated in this article), (4) genes down-regulated in P665 vs. Messire inoculated plants but not between P665 and Messire control plants (called repressed after infection in this article). The number of genes included in each category is included in Fig. 1, and the ID of these genes included in Supplementary Data 1.

Out of the 6780 genes that were more expressed in inoculated P665 compared to inoculated Messire plants, or that were expressed only in inoculated plants in P665, 1498 genes were also more expressed in non-inoculated P665 compared to non-inoculated Messire plants (Fig. 1). Therefore, it can be considered, that the constitutive expression of these genes is higher in P665 than in Messire. By contrast, 2038 genes were more expressed in inoculated P665 compared to inoculated Messire inoculated plants, but not in non-inoculated P665 plants. These genes can be considered, therefore, induced due to the infection in P665.

Regarding the genes down regulated in P665 vs. Messire inoculated plants, 1551 were also less expressed in control plants and can be considered constitutively down regulated in P665 vs. Messire, while 2022 were down-regulated only in inoculated plants and can be considered to be repressed due to the infection.

The proportion of genes included in the different Go-slim categories for each of these groups are included in Supplementary Figs. S3, S4, S5, S6.

**Figure 2:**
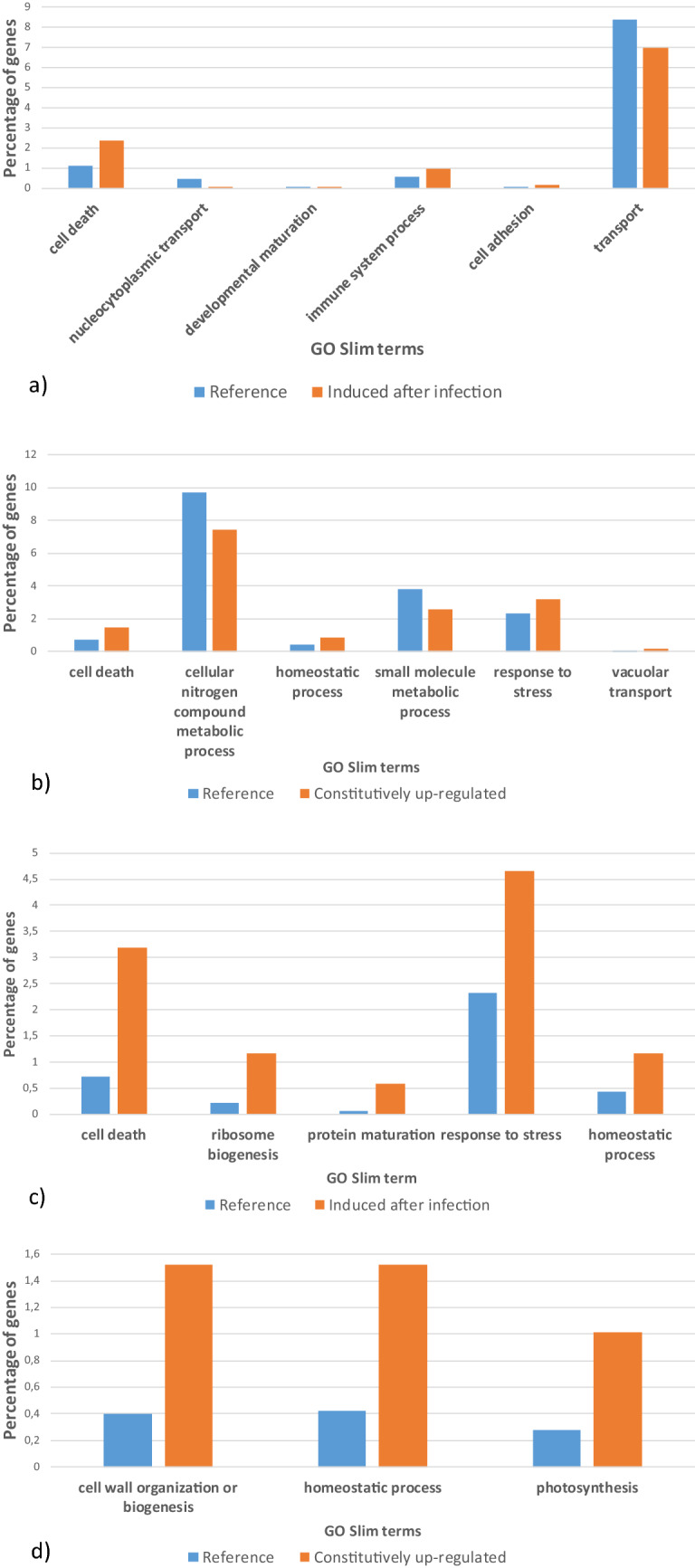
Percentage of transcripts belonging to over/under-represented GO Slim terms in the set of genes (**a**) induced in P665 vs. Messire after infection with *P. pinodes*, (**b**) in the set of transcripts constitutively up-regulated in P665 vs. Messire, (**c**) in the set of genes induced in P665 vs. Messire after infection with *P. pinodes* and in the resistant bulk vs. susceptible bulk, (**d**) in the set of transcripts constitutively up-regulated in P665 vs. Messire and in the resistant bulk vs. susceptible bulk, in comparison to the plant reference created in this study.

Go-enrichment analysis showed that genes induced after infection in P665 vs. Messire were enriched in the biological processes “cell death” and “immune system process”. Other enriched biological processes were “nucleocytoplasmic transport”, “developmental maturation” and “cell adhesion”. Interestingly, these analyses showed that genes constitutively more expressed in P665 vs. Messire were also enriched in biological processes involved in defense as “cell death” and “response to stress” (Fig. 2).

### Identification of genes showing a consistent expressional pattern in resistant vs. susceptible lines

From the RIL population, a total of 566,328,503 sequencing reads were processed after trimming (average 6,024,771 reads per family) (Table 1). From among the genes constitutively up-regulated in P665 vs. Messire, the 12.2% of them (183 transcripts) were also up-regulated between the resistant bulk vs. the susceptible bulk. These genes can be considered consistently constitutively more expressed in resistant than susceptible plants. From among them, 119 were only detected in the resistant bulk libraries (Supplementary Data 2). On the other hand, from the genes induced after infection in P665 vs. Messire, the 16.8% (343 transcripts) can be considered consistently induced in resistant vs. susceptible plants, as they were also more expressed in the resistant bulk vs. the susceptible bulk. From among them, 272 were only detected in the resistant bulk libraries.

From among the genes constitutively down-regulated in P665 vs. Messire, the 7.1% (111 transcripts) were also down-regulated in the resistant bulk vs. the susceptible bulk. These genes can be, therefore, assumed to be consistently constitutively less expressed in resistant than susceptible plants. Among them, 49 were only detected in the susceptible bulk libraries. By contrast, from the transcripts repressed after infection in P665 vs. Messire, the 8.2% (166 transcripts) were less expressed in P665 inoculated vs. Messire inoculated plants, but not in control plants, and also less expressed in the resistant bulk vs. the susceptible bulk, and can be believed to be consistently down-regulated after infection in resistant vs. susceptible plants (Fig. 1).

All these genes, showing a consistent pattern in resistant vs. susceptible plants, could be used as “expression markers” to distinguish between resistant and susceptible plants and are included in Supplementary Data 2.

In conclusion, by applying BSTA strategy, we were able to reduce drastically the number of candidate resistance-related genes (according to their expression in the parental lines) by a minimum of 83.2% (from 92.9 to 83.2% depending on the expression group), identifying consistent expression markers that distinguish resistant from susceptible plants. These results, highlight the power of this approach to identify a set of accurate expression markers useful for breeding.

In the set of genes that were induced after infection in P665 vs. Messire and also more expressed in the resistant bulk *vs* the susceptible bulk, the Go-slims terms “cell death” and “response to stress” were enriched, as well as the Go-terms “ribosome biogenesis”, “protein maturation” and “homeostatic process”. Go-terms enriched in the set of genes constitutively more expressed in P665 vs. Messire, and also more expressed in the resistant Bulk vs. the susceptible bulk were “cell wall organization or biogenesis”, “homeostatic process” and “photosynthesis” (Fig. 2).

### Correlation between the level of expression of the genes and the level of resistance to *P. pinodes*

We could detect the expression of 21,696 transcripts in the RIL population. Of them, expression values in at least 50 RIL families were detected for 13,738 genes (Supplementary Data 3). For these genes, Pearson’s correlation coefficient between the level of expression of the gene in each RIL family and the level of resistance to *P. pinodes* in the families was calculated. Pearson's correlation coefficient ranged between − 0.63 and 0.61. As the level of resistance was scored as the disease rating in a scale from 0 to 5, where 5 corresponded to the most susceptible plants, a negative Pearson's correlation coefficient means that a higher expression of a gene was correlated with resistance, while a positive value indicates that a lower expression of the gene was correlated with resistance. Among the genes showing the highest correlation between a high expression of the gene and a high level of resistance there were several genes having regulatory roles as well as many genes involved in protein synthesis and maturation.

### Validation of expression of relevant genes by qRT-PCR

**Figure 3:**
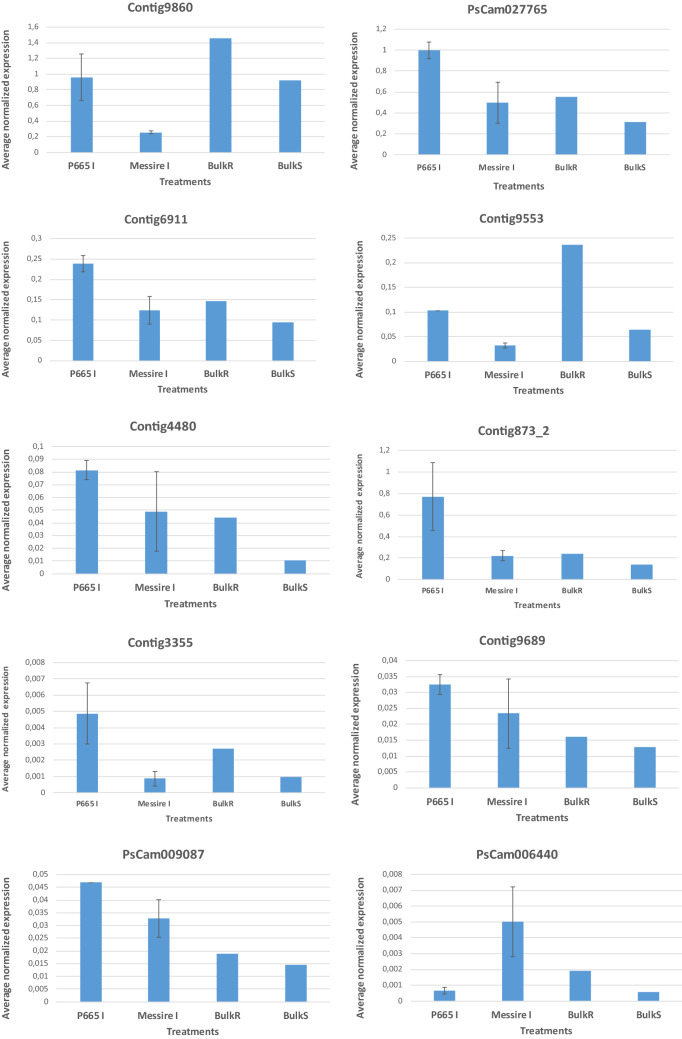
Normalized expression of the different transcripts measured by qRT-PCR. *P665 I* = P665 inoculated plants 24 hai, *Messire* *I* = Messire inoculated plants 24 hai, *BulkR* = bulk of the most resistant RIL families, *BulkS* = bulk of the most susceptible RIL families. Error bars represent standard errors of the means.

Average expression profiles obtained by qRT-PCR showed the same trend as those obtained by MACE. As an exception, in the case of PsCam006440, a higher expression of this gene in the resistance bulk vs. susceptible bulk was confirmed by qRT-PCR, but this gene was only detected in P665 inoculated plant libraries by MACE-Seq, while, by qRT-PCR, it was less expressed in P665 inoculated plants than in Messire inoculated plants (Fig. 3). In addition, in the case of Contig4480 and Contig9689, in the qRT-PCR assay, confirming MACE-Seq results, these genes were more expressed in the resistant bulk than in the susceptible bulk and also in P665 vs. Messire in two of the three replicates performed. However, in the third replicate, these genes were more expressed in Messire than in P665. All these three genes showed a very low expression. This really low expression precludes the accurate measurement of their expression by any method and could explain the discrepancies between MACE-Seq and qRT-PCR results.

## Discussion

Next Generation Sequencing (NGS) techniques are allowing the sequencing of whole genomes and transcriptomes at low cost. NGS transcriptome profiling enable the quantification of differentially expressed genes with high accuracy and has been widely used to study plant-pathogen interactions. While usually by RNA-seq gene expression in only a few genotypes, usually a resistant and a susceptible one, is analysed, we took advantage of the cost efficiency of the MACE-Seq technique to sequence the transcriptome of a whole RIL F_2:6_ population segregating for resistance. This approach allowed us to obtain unprecedented new and valuable information. Thus, we could estimate the level of expression of more than 21,500 transcripts in this population and correlate their expression values with the level of resistance of the different RIL families.

In addition, we performed what we coin “bulked segregant expression analysis” (BSTA) enabling us to reduce the number of candidate resistant-related genes by 83.2 to 92.9% as compared to solely the analysis of the parental lines. Bulked-segregant analysis (BSA) is an efficient method to identify markers tightly linked to genes responsible for a trait^26^. After crossing two parental lines with contrasting phenotypes for the analyzed trait, the derived F_2_ population is evaluated for the trait. Then, two bulks, formed by F_2_ individuals showing contrasting phenotypes are produced and analysed with molecular markers to identify polymorphic markers between the two bulks^27^. The idea behind this method, combined with the power of whole genome sequencing, has been used e.g. for QTL mapping, by whole-genome resequencing of two DNA bulks of progeny showing contrasting phenotype^27^.

We here harnessed the power of NGS in combination with BSA to compare the level of expression of the whole transcriptome in two bulks of families showing contrasting response to *P. pinodes*. Our BSTA approach allowed us to identify the genes contributing more to the observed resistance and, instead of traditional DNA markers linked to resistance, identify, “expression markers”, i.e., genes which expression could be used to distinguish resistant from susceptible lines.

Applying Gene Ontology analysis on this focused set of candidate resistance-related genes enabled a birds-eye view on the data set from the different experimental set-ups revealing key factors for resistance to *P. pinodes.* Especially genes involved in Go-categories “Cell death”, “Response to stress” “Cell wall modification” and “Protein synthesis and maturation” were significantly up-regulated in the resistant parent and RILs and will be discussed in more detail below:

### Cell death has a relevant role in resistance to *P. pinodes*

**Figure 4:**
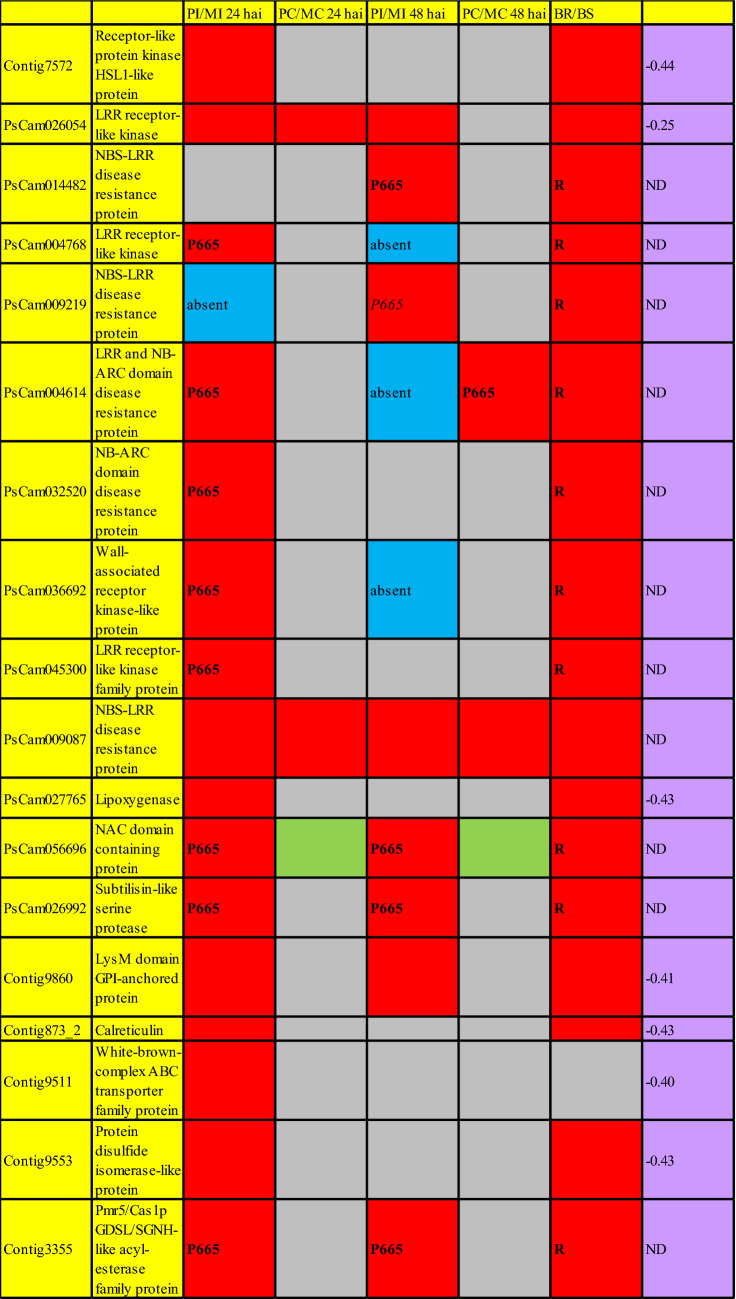
Expression pattern, according to MACE-Seq results, of the most relevant genes with a putative involvement in resistance to *P. pinodes*. Cells in red indicates that the transcript was significantly (log_2_FoldChange > 1; p_adjusted_ ≤ 0.05) up-regulated in P665 compared to Messire. Cells in green indicates that the transcript was significantly (log_2_FoldChange < 1; p_adjusted_ ≤ 0.05) down-regulated in P665 vs. Messire. Cells in grey correspond to genes not differentially expressed between treatments. *P* = P665, *M* = Messire, *I* = Inoculated plants, *C* = control plants, *BR* = Resistant bulk, *BS* = susceptible bulk, *R* = transcript only detected in the resistant bulk, *P665* = transcript only detected in P665, *absent* = transcript absent in these treatments, *hai* = hours after inoculation, *Pearson* = Pearson’s correlation coefficient between the level of the resistance and the expression of the gene, *ND* = not determined.

*P. pinodes* is a hemibiotrophic fungi. Therefore, this pathogen has an initial biotrophic phase during the first stages of the infection that is later on followed by a necrotrophic phase in the mesophyll. Toyoda et al.^28^ already pointed out the relevance of this early biotrophic phase in the pathogenicity of *P. pinodes*. Our previous histological studies^19^, showed that the death of the pea epidermal cell that is infected by *P. pinodes* is a mechanism that contributes to resistance to *P. pinodes*. Hypersensitive response (HR), which is a pathogen-induced cell death process at the site of infection that limits pathogen growth, is a common mechanism of resistance against biothrophic pathogens and our present study corroborates that this mechanism has a relevant role in defense against *P. pinodes*, affecting probably this initial biotrophic phase. Thus, the Go-slim term “cell death” was more frequent in the set of genes up-regulated after infection in P665 vs. Messire and also in the resistant bulk vs. the susceptible bulk. Go-enrichment analysis also showed that in P665 a set of genes involved in this process are constitutively higher expressed compared to the susceptible Messire. HR is the result of the recognition of pathogen effectors by the plant, unleashing effector-triggered immunity (ETI), and is activated by R-genes. Our combined approach identified a set of R-genes and receptor kinases that could have a relevant role in detecting *P. pinodes*, as they were up-regulated after infection, or constitutively more expressed in P665 vs. Messire, and also more expressed in the resistant RIL bulk vs. the susceptible bulk. In addition, their expression was highly correlated with the level of resistance. This set of genes is shown in Fig. 4, and includes transcripts annotated as “LRR receptor kinases”, “NBS-LRR disease resistance proteins”, “NB-ARC domain disease resistance proteins” and a “Wall-associated receptor kinase-like protein”. For one of them, PsCam009087, its consistent higher expression in resistant vs. susceptible plants was confirmed by qRT-PCR.

In addition to R-genes, other up-regulated gene with a putative role in “cell death”, that also showed a good correlation with the level of resistance, was PsCam027765, that encodes a “Lipoxygenase”. The expression profile of this gene was confirmed by qRT-PCR. The function of lipoxygenases (LOX) in the defense has been postulated to be associated with the synthesis of a number of different compounds involved in signaling, having antimicrobial activity or associated with the development of the HR. The HR is characterized by the loss of membrane integrity and closely related to the generation of lipid peroxides and active oxygen species. It has been postulated that LOX-mediated lipid oxidation is important in causing membrane damage during the HR^29^.

### Genes involved in “response to stress” putative contributing to *P. pinodes* resistance

In addition to “cell death”, the Go-slim term “response to stress” was also more abundant in the set of genes consistently induced after infection with *P. pinodes* in resistant vs. susceptible RILs and in P665 vs. Messire. A constitutively higher expression of genes associated with defence in P665 vs. Messire had been already reported in our previous transcriptome studies^10,11^. Several genes had a putative involvement in defense, according to their annotation, and meet the requirements of being up-regulated after infection, or constitutively more expressed in P665 vs. Messire, and also more expressed in the resistant RIL bulk vs. the susceptible bulk, being also for many of them their expression correlated with the level of resistance. These genes are shown in Fig. 4.

The possible role of these genes in resistance to *P. pinodes* in pea is discussed below. Thus, a gene putative involved in defense to *P. pinodes* according to our study is PsCam056696, encoding a protein containing a NAC domain. Expression of this genes was only detected in P665 inoculated plants and in the libraries obtained from the resistant bulk, suggesting that its expression contributes to resistance. Several studies have revealed that a number of NAC TFs acts as regulators of plant immunity, modulating the hypersensitive responses and stomatal immunity or acting as virulence targets of pathogen effectors (for a review see Yuan et al.^30^).

Another remarkable gene with a putative interesting role in *P. pinodes* resistance is PsCam026992, encoding a “Subtilisin-like serine protease”. This gene was more expressed in P665 than in Messire in both inoculated and control plants. Regarding the expression in the bulks, expression of this gene was only detected in the resistant bulk. There are several evidences about the role of Subtilisin-like serine proteases (also called subtilases) in pathogen interactions. Thus, several subtilisin-like proteases are involved in plant resistance against pathogens, they are secreted to the plant extracellular matrix and may play relevant roles in pathogen recognition and subsequent signaling cascades resulting in the expression of genes acting in defense. In addition, some sub-groups of the subtilase have a relevant role in programmed cell death. They have been also suggested to be associated to immune priming in plants (all these subtilase features are reviewed in Figueiredo et al.^31^).

Another gene that was up-regulated after infection in P665 compared to Messire, being also up-regulated in the resistant bulk compared to the susceptible bulk is “Contig9860”. This expression profile was confirmed by qRT-PCR and, in addition, a higher expression of this gene was highly correlated with resistance. This gene was predicted to encode a “LysM domain GPI-anchored protein”. Plants notice the presence of pathogens through the detection of microbe-associated molecular patterns (MAMPs) activating the so-called MAMP-triggered response. Chitin is a representative MAMP molecule from fungi and induces immune responses in many plant species^32^. Several studies had shown that lysin motif (LysM)-containing proteins are essential for plant recognition of chitin, leading to the activation of plant innate immunity^33,34^. Therefore, we postulate that Contig9860 could detect the chitin of *P. pinodes* thereby activating plant defenses.

Contig873_2, encoding a “Calreticulin” protein was induced after infection in P665 vs. Messire, and was also more expressed in the resistant bulk vs. susceptible bulk. A higher expression of this gene was, in addition, correlated with the level of resistance. Recent studies suggest that Calreticulin (CRT) isoforms are important regulators of plant immunity^35^. While in arabidopsis *atcrt1* and *atcrt2* mutant plants are more resistant to *Pseudomonas syringae* pv *tomato* infection than wild-type plants, *atcrt3* mutants are more sensitive. AtCRT3 is indispensable for the abundance of functional leucine-rich repeat receptor kinase EFR. EFR specifically recognize bacterial elongation factor (EF)-Tu and its surrogate peptide elf18, inciting the initiation of pathogen-associated molecular pattern-triggered immunity (PTI)^35^. The CRT isoform having a higher similarity with Contig873_2 is AtCRT3. Contig873_2 could have, therefore, a role in perceiving *P. pinodes* and trigger plant defenses. A further evidence suggesting a role of CRT in plant-pathogen interaction is that CRT were detected in a search for oligogalacturonide-modulated phosphoproteins. Oligogalacturonides are elicitors of the plant defense reaction to pathogens, being the observation that CRT is phosphorylated during this process interesting. Furthermore, in tobacco CRT mRNA transcript levels were elevated following treatment of plants with a range of signaling molecules involved in the response to pathogen attack^36^.

### Cell wall modifications also contributes to resistance to *P. pinodes*

A gene (Contig3355) that was only detected in P665 inoculated plants, and also only in the resistant bulk libraries in our MACE-Seq experiment, encoded a “Pmr5/Cas1p GDSL/SGNH-like acyl-esterase family protein”. This expression profile was also confirmed by qRT-PCR, where this gene was up-regulated in P665 inoculated plants at 24 hai compared to Messire and in the resistant bulk compared to the susceptible bulk. PMR5 is part of the DUF231 family that contains cell wall-modifying enzymes. Chiniquy et al.^37^ provided evidences suggesting that PMR5 is a functional acetyltransferase that mediates pectin acetylation on GalA residues, and this leads to altered pathogen resistance. These authors found that *pmr5* mutant were more susceptible to the necrotrophic pathogen *Botrytis cinerea*, suggesting that PMR5 could have a role in resistance to necrotrophic pathogens. *pmr5* mutants showed a decrease in cellulose content, facilitating cell wall hydrolysis by the fungus. By contrast, PMR5 reinforced cell walls and reduced their hydrolysis by *P. pinodes*. In addition, in *Arabidopsis* PMR5 contributed to PEN2-mediated preinvasion resistance to the hemibiotrophic fungus *Magnaporthe oryzae*^38^. Therefore, Contig3355 could contribute to reinforce P665 cell wall making it more difficult to be degraded by *P. pinodes*.

Another gene with a putative involvement in cell wall formation is Contig9511. This gene was more expressed in P665 inoculated plants at both 24 and 48 hai, compared to Messire and also in the resistant bulk compared to the susceptible one. The expression profile of this gene was also confirmed by qRT-PCR and a higher expression of this gene was associated with a higher resistance in the RIL population. This gene was predicted to encode a “White-brown-complex ABC transporter family protein”. This kind of proteins have been reported to be involved in cutin and maybe wax transport to the extracellular matrix and may also play a vital role in stress responses. Cuticle functions includes protection from environmental stresses and pathogens. The major component of the cuticle is cutin and embedded in the cutin matrix are cuticular waxes^39^. Contig9511 could have a role in repairing the cell wall damage caused by *P. pinodes* infection.

### Expression of genes involved in protein synthesis and maturation were highly correlated with the level of resistance to *P. pinodes*

From among the genes which expression was more correlated to the level of resistance to *P. pinodes* there were many involved in protein synthesis and maturation. In agreement with that, the Go-slim term “ribosome biogenesis” was more abundant in the set of genes induced after infection. Genes showing an expression highly correlated with resistance included a number of ribosomal proteins, histones, chaperones, and proteins involved in protein maturation. Particularly, we confirmed the expression profile of one these genes, Contig9553, by qRT-PCR. This gene encodes a “disulfite isomerase” and was induced after infection in P665 vs. Messire, 24 hai and also more expressed in the resistant bulk vs. the susceptible bulk. Its expression was also highly correlated with the level of resistance. Protein disulfide isomerase, is an enzyme that catalyzes the formation and breakage of disulfide bonds between cysteine residues within proteins as they fold. This allows proteins to quickly find the correct arrangement of disulfide bonds in their fully folded state, and, therefore, the enzyme acts to catalyze protein folding^40^. A proteomic study performed by us also found that ribosomal proteins expression significantly increased in response to *P. pinodes*^41^. These evidences reveal the active reprograming of P665 metabolism to fight against *P. pinodes*.

## Conclusions

In our study whole transcriptome profiling by MACE-Seq revealed a complex pattern of significantly up-and down-regulated genes in resistant vs. susceptible plants. From among them, constitutively or infection-related genes up-regulated only in the resistant plants, are of particular interest and offer itself for a more detailed analysis. However, usually, and also in our case, thousands of genes may fulfill this criterion, if only the narrow genetic context of two contrasting plants is analysed. Thus, filtering is needed to tame the data flood and end up in a handsome number of marker genes suitable for functional studies and MAS in breeding. The BSTA approach we present here may provide such a filter, as it enabled us to identify those marker genes that were consistently related to resistance over generations in a controlled broader genetic context provided by the RILs. Thus, our study provides a novel, generally applicable approach for the economically and efficient application of NGS for the identification of expression markers for MAS in breeding.

## Supplementary Information

Supplementary Figure S1.

Supplementary Figure S2.

Supplementary Figure S3.

Supplementary Figure S4.

Supplementary Figure S5.

Supplementary Figure S6.

Supplementary Information 1.

Supplementary Information 2.

Supplementary Information 3.

Supplementary Table S1.

## Data Availability

The datasets generated and/or analysed during the current study are available in the GEO repository (accession number GSE206312).
