# Supplementary material for: Bulked segregant transcriptome analysis in pea identifies key expression markers for resistance to Peyronellaea pinodes
Source: Sci Rep. 2022 Oct 28;12:18159. doi: 10.1038/s41598-022-22621-2 (PMC9616913; doi:10.1038/s41598-022-22621-2)
Supplement: Supplementary file 1 — Supplementary Figure S1. [file 41598_2022_22621_MOESM1_ESM.pptx]

## Slide 1
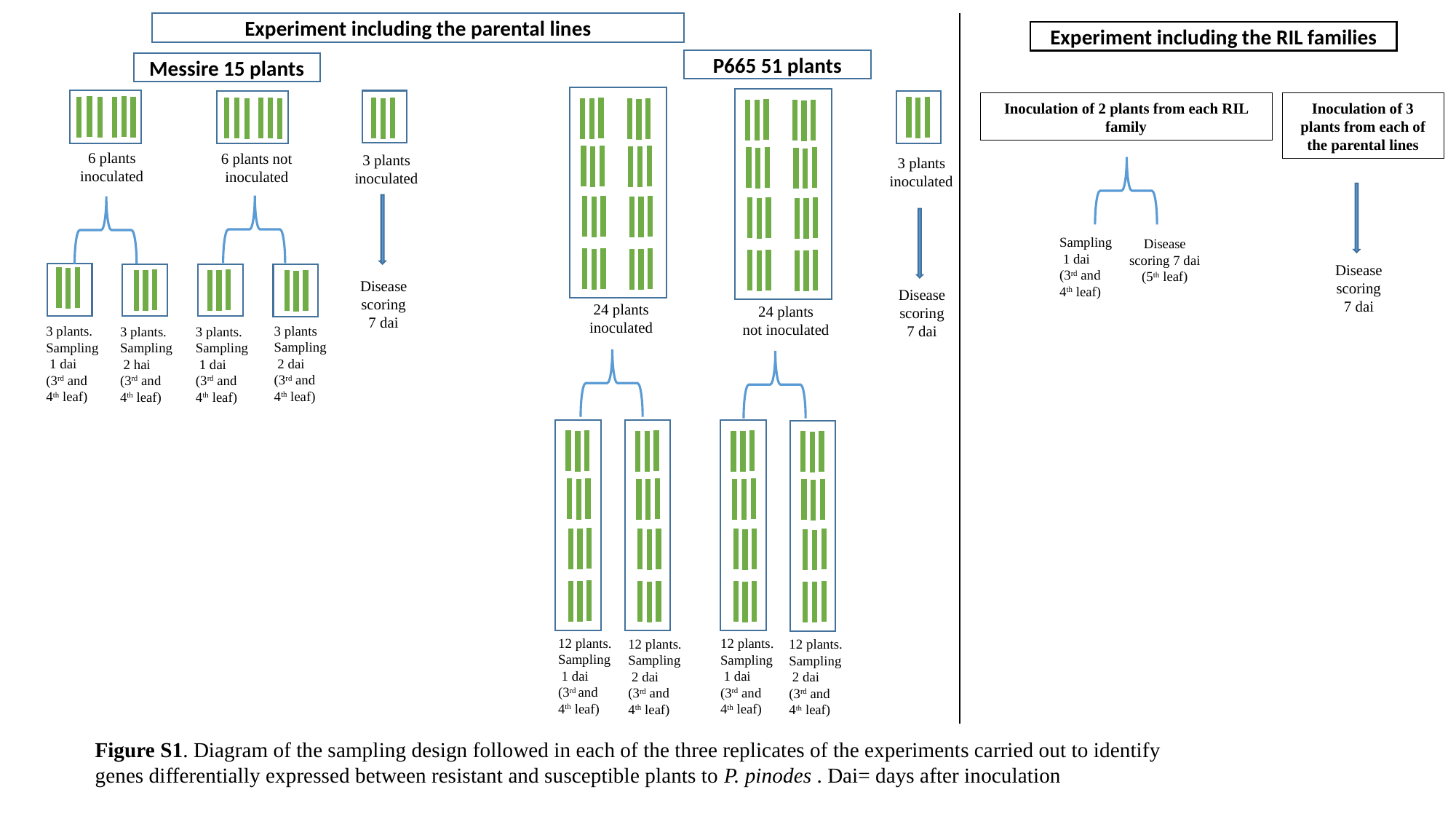

Experiment including the parental lines
Experiment including the RIL families
P665 51 plants
Messire 15 plants
Inoculation of 2 plants from each RIL family
Inoculation of 3 plants from each of the parental lines
6 plants
inoculated
6 plants not inoculated
3 plants
inoculated
3 plants
inoculated
Sampling
 1 dai
(3rd and
4th leaf)
Disease scoring 7 dai (5th leaf)
Disease scoring 7 dai
Disease scoring 7 dai
Disease scoring 7 dai
24 plants
inoculated
24 plants
not inoculated
3 plants Sampling
 2 dai
(3rd and
4th leaf)
3 plants. Sampling
 1 dai
(3rd and
4th leaf)
3 plants. Sampling
 1 dai
(3rd and
4th leaf)
3 plants. Sampling
 2 hai
(3rd and
4th leaf)
12 plants. Sampling
 1 dai
(3rd and
4th leaf)
12 plants. Sampling
 1 dai
(3rd and
4th leaf)
12 plants. Sampling
 2 dai
(3rd and
4th leaf)
12 plants. Sampling
 2 dai
(3rd and
4th leaf)
Figure S1. Diagram of the sampling design followed in each of the three replicates of the experiments carried out to identify genes differentially expressed between resistant and susceptible plants to P. pinodes . Dai= days after inoculation
