# Supplementary material for: Bulked segregant transcriptome analysis in pea identifies key expression markers for resistance to Peyronellaea pinodes
Source: Sci Rep. 2022 Oct 28;12:18159. doi: 10.1038/s41598-022-22621-2 (PMC9616913; doi:10.1038/s41598-022-22621-2)
Supplement: Supplementary file 2 — Supplementary Figure S2. [file 41598_2022_22621_MOESM2_ESM.docx]

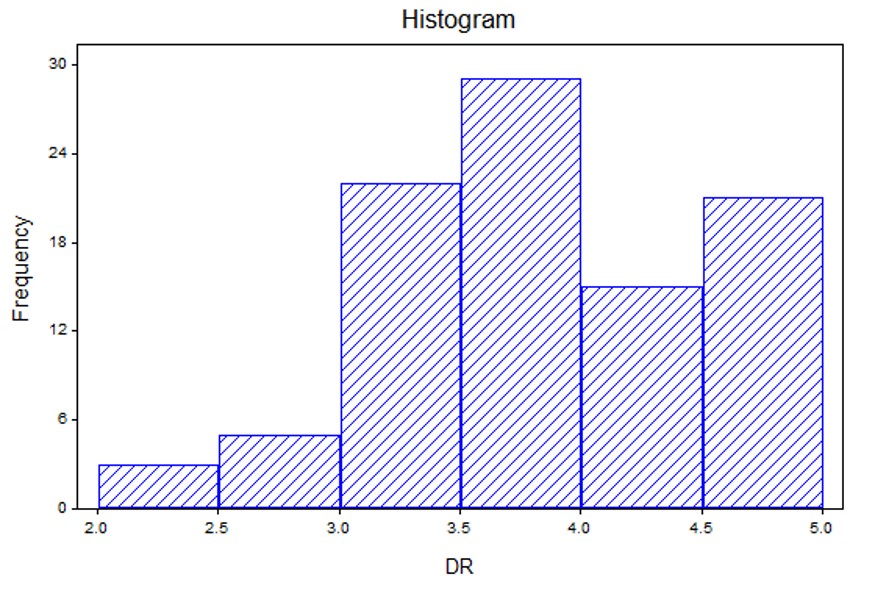


**Figure S2.** Histogram of disease rating (DR) (Roger and Tivoli, 1996) obtained one week after inoculation with *P. pinodes* in the RIL population P665 x Messire
