# Supplementary material for: Bulked segregant transcriptome analysis in pea identifies key expression markers for resistance to Peyronellaea pinodes
Source: Sci Rep. 2022 Oct 28;12:18159. doi: 10.1038/s41598-022-22621-2 (PMC9616913; doi:10.1038/s41598-022-22621-2)
Supplement: Supplementary file 5 — Supplementary Figure S5. [file 41598_2022_22621_MOESM5_ESM.docx]

A)

B)

**Figure S5.** Percentage of transcripts belonging to each GO Slim term for the GO categories (a) Biological process and (b) Molecular function, in the set of genes repressed after infection in P665 *vs* Messire. Only the 25 more represented Go terms are shown. The general Go Slims categories “biological process” and “molecular function” were excluded.
