# Supplementary material for: Bulked segregant transcriptome analysis in pea identifies key expression markers for resistance to Peyronellaea pinodes
Source: Sci Rep. 2022 Oct 28;12:18159. doi: 10.1038/s41598-022-22621-2 (PMC9616913; doi:10.1038/s41598-022-22621-2)
Supplement: Supplementary file 10 — Supplementary Table S1. [file 41598_2022_22621_MOESM10_ESM.docx]

| **Transcript** | **Annotation** | **Forward primer (5´-3´)** | **Reverse primer (5´-3´)** |
| --- | --- | --- | --- |
| Contig335 | Pmr5/Cas1p GDSL/SGNH-like acyl-esterase family protein | aaagaatacaatgcaaccattgag | ggatcatccgagtttgattctact |
| Contig4480 | Cellulose synthase-like protein | gagctgggatttgcagtctt | ttttccatgcacataatcatagc |
| Contig9689 | Transducin/WD40 repeat protein | aagacggatggacactgctc | tgaagatcccacaatgttacaatc |
| PsCam009087 | NBS-LRR disease resistance protein | actcgtcttaagcgcctttg | tgatggaagtcacgtggaag |
| PsCam027765 | Lipoxygenase | tttgtgtctccttcgtgcat | cggctttcacattacgagatt |
| Contig6911 | Cytochrome P450 family Ent-kaurenoic acid oxidase | ggataccgaattgggaggata | ttgctatccatgttgcatcc |
| Contig9860 | LysM domain GPI-anchored protein | aacacttcaagccggtcaag | tggaacaagcaatggaaaatc |
| Contig9553 | Protein disulfide isomerase-like protein | agttacaaaaatgacggaaatgaa | caaagacaaaaacctggtgct |
| Contig873_2 | Calreticulin | gatgaactttgaaatgggcaat | ctgcaacatcaattatgcagtg |
| PsCam006440 | NB-ARC domain disease resistance protein | tttggatcctcaaaggcaat | cgacccgacacctacgaa |

**Supplementary Table 1.** Primer sequences for the amplification by qRT-PCR
